# Perforins Expression by Cutaneous Gamma Delta T Cells

**DOI:** 10.3389/fimmu.2020.01839

**Published:** 2020-08-14

**Authors:** Katelyn O'Neill, Irena Pastar, Marjana Tomic-Canic, Natasa Strbo

**Affiliations:** ^1^Department of Microbiology and Immunology, Miller School of Medicine, University of Miami, Miami, FL, United States; ^2^Wound Healing and Regenerative Medicine Research Program, Dr. Phillip Frost Department of Dermatology and Cutaneous Surgery, Miller School of Medicine, University of Miami, Miami, FL, United States

**Keywords:** pore forming proteins, perforin, perforin-2, mpeg-1, gamma delta T cells, skin, cytotoxicity

## Abstract

Gamma delta (GD) T cells are an unconventional T cell type present in both the epidermis and the dermis of human skin. They are critical to regulating skin inflammation, wound healing, and anti-microbial defense. Similar to CD8+ cytotoxic T cells expressing an alpha beta (AB) TCR, GD T cells have cytolytic capabilities. They play an important role in elimination of cutaneous tumors and virally infected cells and have also been implicated in pathogenicity of several autoimmune diseases. T cell cytotoxicity is associated with the expression of the pore forming protein Perforin. Perforin is an innate immune protein containing a membrane attack complex perforin-like (MACPF) domain and functions by forming pores in the membranes of target cells, which allow granzymes and reactive oxygen species to enter the cells and destroy them. Perforin-2, encoded by the gene *MPEG1*, is a newly discovered member of this protein family that is critical for clearance of intracellular bacteria. Cutaneous GD T cells express both Perforin and Perforin-2, but many questions remain regarding the role that these proteins play in GD T cell mediated cytotoxicity against tumors and bacterial pathogens. Here, we review what is known about Perforin expression by skin GD T cells and the mechanisms that contribute to Perforin activation.

## Introduction

Gamma delta (GD) T cells are an unconventional T cell type that constitutes about 1–5% of circulating lymphocytes (1, 2). Despite low numbers in circulation, GD T cells are enriched in barrier tissues including the skin, gut, and reproductive tract (3–8). The skin is an epithelial tissue that serves as a barrier to protect against physical and chemical insults as well as potentially pathogenic microorganisms. It is composed of two main layers, the epidermis and dermis, and GD T cells are present in both layers in both mice and humans (2, 9). Epidermal GD T cells in mice are referred to as dendritic epidermal T cells (DETCs) because of the dendritic processes they use to survey surrounding keratinocytes for signs of stress or damage (10, 11). These cells all express an identical invariant TCR and are potent producers of IFN-g (12–14). Dermal GD T cells on the other hand are not dendritic and they produce IL-17 (5, 6). Human GD T cells, however, exist primarily in the dermis (15, 16). Small numbers are observed in the epidermis in steady state, but unlike mouse DETCs, they do not exhibit dendritic processes (17). Human GD T cells also express an invariant TCR. Skin resident cells express a delta 1 TCR, while circulating GD T cells express a delta 2 TCR (18–20).

In recent years, researchers have begun to elucidate the vast roles that GD T cells play in skin inflammation and anti-microbial defense. GD T cells serve as bridges between the innate and adaptive immune system. Although they express a recombinant TCR as alpha beta (AB) T cells do, they can bind to and recognize antigens directly without processing and presentation by MHC molecules (21). They can also respond to antigens without the need for pre-stimulation (6, 22). These innate-like characteristics position GD T cells to respond quickly to signs of stress caused by damage or infection. GD T cells play an important role in the response to pathogens in the skin. They produce IL-17 and IL-22 upon stimulation with IL-23, which triggers antimicrobial peptide production by keratinocytes as well as neutrophil recruitment to the site of infection (5, 9, 23). GD T cells also contribute to wound healing, particularly through their production of fibroblast growth factor-7 (FGF-7) and insulin-like growth factor-1 (IGF-1), which promote keratinocyte proliferation and reepithelialization (24, 25), as well as through their production of IL-17 (26). They also secrete fibroblast growth factor-9 (FGF-9), which triggers Wnt activation in wound fibroblasts and modulates hair follicle regeneration after wounding (27). In addition, GD T cells play a role in tumor surveillance. They have the ability to kill a variety of cutaneous tumors including melanoma and carcinomas, and they express cytotoxic molecules including Perforins and granzymes (18, 28, 29). Although researchers have documented Perforin production by GD T cells, the mechanisms regulating Perforin production and the pathways through which it stimulates GD T cell cytotoxicity are not fully understood. This review will cover what is known about the role of Perforin as well as the novel pore forming protein Perforin-2 in GD T cells in the skin.

## Overview of the Perforins

Some of the most well-characterized pore forming proteins include the pore forming toxins expressed by pathogenic bacteria (30). One example is the cholesterol dependent cytolysins (CDC) which are produced by several gram-positive bacterial species. These virulence factors promote bacterial pathogenesis by lysing or permeabilizing host cell membranes or intracellular organelles (30–32). CDC proteins share a complex core fold with proteins from the membrane attack complex/perforin (MACPF) superfamily (33–35). This structural homology underlies the similar mechanism of pore formation and membrane disruption shared by both protein families. Pore forming proteins in the MACPF family are named as such because they all contain a domain that is shared by the proteins that form the membrane attack complex and the Perforins (36). Hundreds of MACPF domain- containing proteins have been identified, but some of the most well-characterized are the mammalian MACPF immune proteins, which include complement proteins C6-C9, Perforin, and Perforin-2 (34, 37, 38). Perforin, encoded by the gene *PRF1*, is located within cytolytic granules inside cytotoxic T cells and natural killer (NK) cells (39). When the cytotoxic cell recognizes a transformed or infected target cell, the granules containing Perforin, granzymes, and granulysin migrate to the cell membrane and release their contents into the immune synapse (40). Perforin binds to the plasma membrane of the target cell and forms pores in the cell membrane, allowing delivery of cytolytic effector proteins and subsequent destruction of the cell (39–41). Perforin-2, encoded by the gene *MPEG1*, is a recently discovered innate immune protein that is highly conserved throughout the animal kingdom (42–44). Perforin-2 is the more ancient of the two Perforins and it is thought that Perforin originated as a gene duplication of *MPEG1* (45). Perforin-2 differs from other MACPF pore formers in that it has a transmembrane domain and localizes to endosomal membranes. The Perforin-2 cytosolic tail directs the endosomes to bacteria- encapsulating phagosomes (46). Acidification of the phagosome stimulates reconfiguration of the MACPF domain, resulting in pore formation on the bacterial cell membrane (46, 47). Our group was the first to demonstrate the essential role of Perforin-2 in eliminating intracellular bacterial infections (48, 49), confirming the importance of this protein as an antimicrobial effector protein expressed by both phagocytic and tissue forming cells.

## Perforin Expression by Skin GD T Cells

**Figure 1:**
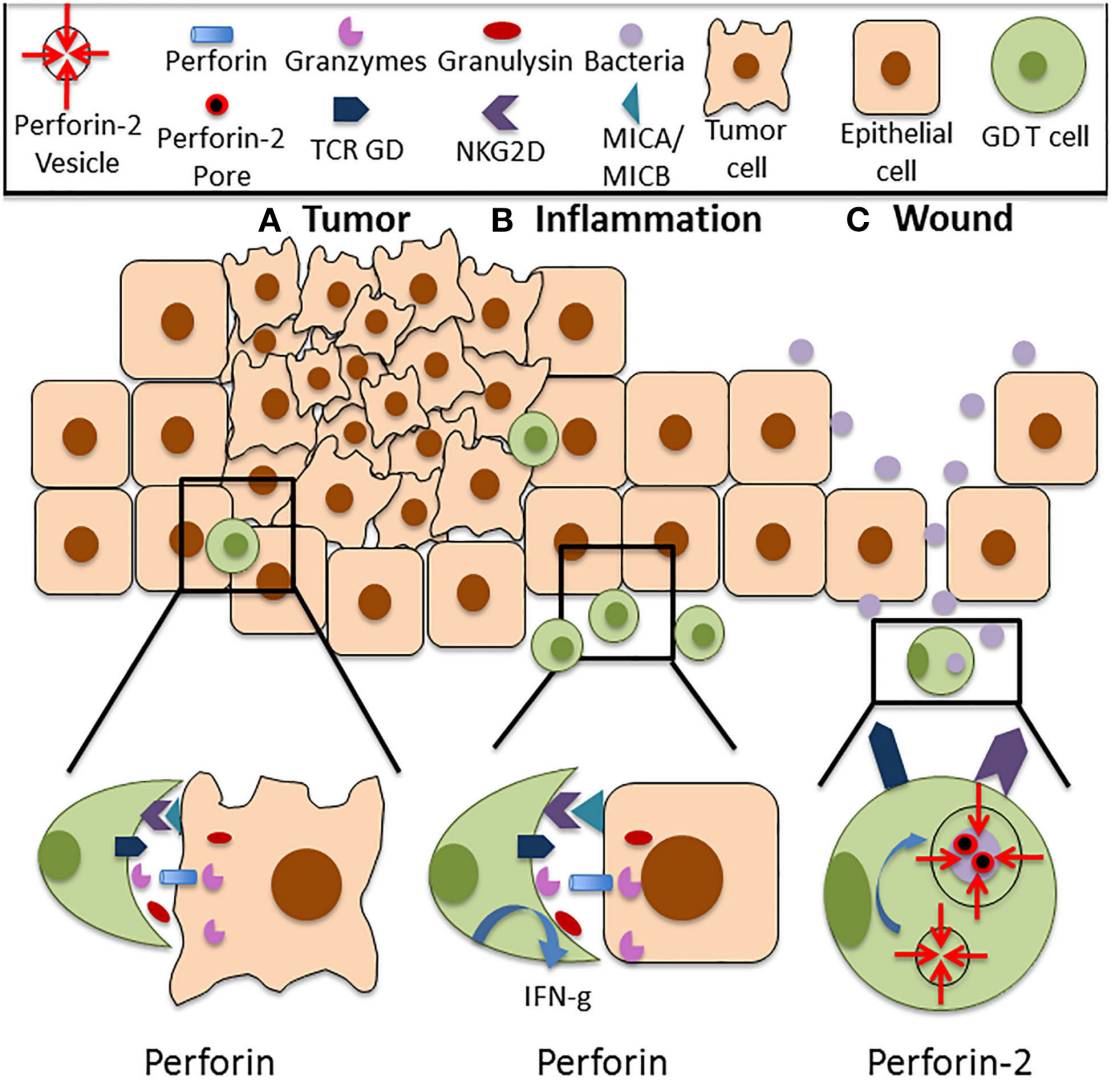
Functions of Perforin in cutaneous GD T cells. **(A)** Cutaneous GD T cells exhibit cytotoxicity against an array of tumor cell types, and this is associated with Perforin expression both at the mRNA and protein level. Perforin is located within cytolytic granules inside cytotoxic GD T cells and they are released upon degranulation into the immune synapse. Perforin binds to the plasma membrane of the target cell and forms pores in the cell membrane, allowing granzymes, granulysin, and reactive oxygen species to enter the cell and destroy it. Cytotoxic GD T cells can become activated through TCR stimulation or through ligation of several costimulatory surface molecules, particularly NKG2D. NKG2D recognizes the stress induced ligands MICA and MICB, and NKG2D signaling is sufficient for activation of skin GD T cell cytotoxicity. **(B)** Perforin expressing GD T cells are also implicated in autoimmune and inflammatory skin diseases. Increased percentages of GD T cells have been observed in the skin of patients with systemic sclerosis, pemphigus vulgaris, Behcet's disease, and psoriasis. These cells express Perforin and granzymes and demonstrate enhanced cytotoxicity in comparison to cells from healthy controls. They also exhibit increased IFN-g expression. **(C)** Cutaneous GD T cells also express the newly discovered innate immune protein Perforin-2. Unlike other MACPF pore formers, Perforin-2 localizes to endosomes that fuse with the phagosome upon intracellular bacterial infection, facilitating pore formation on the bacterial cell membrane. Perforin-2 is essential for the elimination of intracellular bacteria. Given the established role of GD T cells in the antimicrobial response, it is likely that damage to the skin barrier and bacterial entry into the skin contribute to Perforin-2 induction in these immune surveillance cells.

The tumor-lysing capabilities of GD T cells have been well-documented in human skin (Figure 1A). Human skin derived GD T cells were purified using single cell sorting and tested in cytotoxicity assays against a variety of melanoma cell lines. They demonstrated cytotoxicity against SK-Mel2 and HS-294 melanoma cells, resulting in up to 90% cell death. This was comparable to the cytotoxic activity of the CD8+ AB T cells and NK cells that were also tested (18). GD T cells, CD8+ AB T cells, and NK cells only expressed Perforin after being cultured in the presence of IL-2, which is a previously established mechanism of Perforin induction in cytotoxic CD8+ T cells (18, 50, 51). Murine cutaneous Vdelta1+ GD T cells also express Perforin both at the mRNA and protein levels (51). They exhibited cytotoxicity against several tumor cell lines and also expressed granzyme B in amounts comparable to cytotoxic CD8+ AB T cells. Cytotoxic GD and AB T cells both produced IFN-g and TNF-a (18, 52, 53). Additionally, increased numbers of circulating CD3+TCR GD+ cells were observed in melanoma patients in comparison to healthy controls. These cells highly expressed Perforin in both normal individuals and melanoma patients, which may be important to anticancer surveillance (54). However, a study using a mouse model of skin carcinoma reported that circulating IL-17 producing GD T cells supported cutaneous tumor progression by promoting angiogenesis (55). In contrast to cytotoxic skin resident GD T cells, these non-skin resident IL-17 producing GD T cells that infiltrated the skin after tumor formation expressed low levels of Perforin and increased levels of the tumor-promoting factor COX-2. Although this paper did not establish a causative link between reduced Perforin expression and IL-17 production by circulating GD T cells, it implies that low levels of Perforin in these cells may contribute to their lack of cytotoxic activity and allow them to acquire a pro-tumor GD T cell phenotype. These results underscore the importance of Perforin as an effector molecule in GD T cell mediated cytotoxicity in the skin.

Despite the clear importance of Perforin expressing cytotoxic GD T cells in the cutaneous anti-tumor response, these cells have the potential to develop into aggressive T cell lymphomas (56, 57). Primary cutaneous GD T cell lymphomas constitute a subgroup of aggressive T cell lymphomas that express a mature cytotoxic phenotype. These tumors are characterized by their expression of T-cell-restricted intracellular antigen-1 (TIA-1), granzyme B, and Perforin (56–58) and there is also a strong correlation between CD30 expression and cytotoxic protein production (59). Cutaneous GD T cell lymphomas, while rare, have a poor prognosis; therefore, appropriate tumor phenotyping could be a useful tool for diagnosing this disease.

Perforin expressing GD T cells are also implicated in autoimmune and inflammatory skin diseases (Table 1). Patients with the autoimmune disease systemic sclerosis exhibit an increased percentage of Vdelta 1+ GD T cells that demonstrate cytotoxic activity in the skin (69, 70). Increased percentages of CD27+ GD T cells expressing Perforin and granzyme B are also present (71). Patients with Pemphigus vulgaris, an autoimmune disease that causes skin blistering, also have an increased percentage of circulating GD T cells. These cells demonstrate increased activation and cytotoxic activity in comparison to healthy controls (72). Behcet's disease, a disorder that causes blood vessel inflammation, is also associated with the expansion of GD T cells exhibiting increased Perforin and granzyme A expression (73, 74). Increased Perforin and granzyme B expression by CD3+ T cells is also evident in the epidermis of psoriasis patients (75–77). Only the CD8+ and CD4+ T cell subsets were analyzed, so Perforin expression by GD T cells in psoriasis has not yet been confirmed. It has been confirmed, however, that dermal GD T cells are recruited to the epidermis in psoriasis by activated keratinocytes that produce the chemokines CCL2 and CCL20 (78). Recruited dermal GD T cells induce an innate-like immune response by producing IL-17 and IL-22 upon IL-1 and IL-23 stimulation. IL-1 is expressed by keratinocytes while IL-23 is expressed by dermal dendritic cells and macrophages. IL-17 and IL-22 production further drives keratinocyte hyperplasia, neutrophil recruitment and disease progression (5). Since pro-inflammatory GD T cells are recruited to psoriatic skin, they are likely to be among the Perforin expressing CD3+ T cells observed in psoriasis patients (Figure 1B). Overall, studies have shown that GD T cells in the skin express Perforin, and this is associated with an activated cytotoxic phenotype. This can contribute positively to skin immune function via induction of the anti-tumor immune response, or it may have deleterious effects in the case of autoimmune and inflammatory skin disorders or cutaneous GD T cell lymphomas.

**Table 1:** Perforins expressed by cutaneous GD T cells.

	**Perforin**	**Perforin-2**
Location in the cell	Intracellular cytolytic granules (39)	Membranes of intracellular endosomes (46, 47)
Function in GD T cells	Lysis of transformed or infected cells (39–41)	Destruction of intracellular bacteria (46, 47)
Stimulatory factors	Surface receptors: - GD TCR (53) - NKG2D (13, 18, 53, 60) - 2B4 (61) - B7-1 (62) Cytokines: - IL-2 (61) - IL-12 (63) - IL-15 (63) Stress induced molecules: - Hsp60 (64) - Hsp70 (64) Transcription Factors: - IRF-1 (63) Therapeutics: - Rapamycin (65)	Commensal bacteria: - *S. epidermidis* (66)
Inhibitory factors	Surface receptors: - Ly49 (67) - CD94 (67)	Pathogenic bacteria: - *S. aureus* (68)
Role in disease	Expressed by cutaneous GD T cell lymphomas (56–59) Upregulated in autoimmune and inflammatory diseases: - Systemic sclerosis (69–71) - Pemphigus vulgaris (72) - Behcet's disease (73, 74) - Psoriasis (75–77)	Upregulated in response to wounding (68) Downregulation may contribute to persistent wound infection (68)

*Summary of the information currently known about Perforin and Perforin-2 expression by cutaneous GD T cells. Listed are the location of the Perforins within the cell, functions of the Perforins in GD T cells, Perforin stimulatory and inhibitory factors, and the role of Perforins in cutaneous diseases*.

## Mechanisms Regulating Perforin Expression in Skin GD T Cells

Although it is clear that cutaneous cytotoxic GD T cells express Perforin, the mechanisms that regulate its expression are still unclear. Perforin is copiously expressed once these cells become activated (18). In contrast to CD8+ AB T cells which require TCR binding to MHC for activation (79), cytotoxic GD T cells can become activated through a non-MHC-restricted mechanism (61). In fact, recognition and killing of target cells can proceed through ligation of several costimulatory surface molecules in the absence of TCR stimulation (53). One of these surface molecules is the activating NK receptor NKG2D. It recognizes the MHC class I polypeptide-related sequence A and B (MICA and MICB), which are induced on stressed cells (53). NKG2D is expressed by most NK and CD8+ AB T cells, but it was found that both mouse and human GD T cells constitutively express NKG2D as well (53, 80, 81). Its expression is maintained during cell culture of freshly isolated GD T cells (13, 18, 53). NKG2D ligation stimulates epidermal GD T cell degranulation using a phosphatidylinositol 3-kinase (PI3K)–dependent pathway, and killing of NKG2DL expressing target cells occurs in the absence of CD3 and TCR signaling (60). Treatment of skin GD T cells with anti-NKG2D antibodies impaired lysis of A20 B cell lymphoma cells, but treatment with anti-TCR antibodies had no effect, indicating that NKG2D signaling is sufficient to activate cytotoxicity in skin GD T cells (53). Skin GD T cells also express the surface glycoprotein 2B4. Removal of IL-2 from GD T cell culture media reduced surface expression of 2B4 and also reduced their capacity to lyse target cells (61). Their cytotoxicity was enhanced by treatment with a stimulatory anti-2B4 antibody, indicating the importance of this receptor in cutaneous GD T cell mediated cytotoxicity. B7-1 (CD80) also provides costimulatory signals to both AB and GD T cells by binding to CD28 on the T cell surface, increasing adhesion to the target cell. B7-1 surface expression on Pam212, murine squamous cell carcinoma cells, stimulated cutaneous GD T cell proliferation and increased carcinoma cell sensitivity to GD T cell mediated lysis (62). Cutaneous GD T cell cytotoxicity is regulated through expression of several inhibitory receptors that block cytotoxicity upon binding with their appropriate ligands. Murine skin GD T cells express the inhibitory receptors Ly49 and CD94/NKG2. Presentation with Qdm, the ligand for CD94/NKG2, prevented the epidermal GD T cells from killing target cells (67).

GD T cell cytotoxicity is also activated by their recognition of stress-induced molecules on target cell surfaces. Macrophages and neutrophils externalize the heat shock proteins Hsp60 and Hsp70 under inflammatory conditions to target them for destruction, thus resolving the inflammation and preventing excess tissue damage. Cytotoxic GD T cells recognize these molecules and kill the target cells via direct cell-cell interactions (64). Activation of Perforin expressing cytotoxic GD T cells is also mediated through cytokine expression. IL-2, IL-15, and IL-12 enhance cytolytic activity of cutaneous GD T cells, and the transcription factor IFN regulatory factor-1 (IRF-1) is essential for induction of cytotoxicity (61, 63). The Perforin dependent anti-tumor properties of GD T cells can be enhanced pharmacologically. For example, rapamycin enhances the perforin-dependent cytotoxicity of human GD T cells against squamous cell carcinomas *in vitro* and in a mouse xenograft model of human squamous cell carcinoma (65). Additionally, Resveratrol enhances Perforin expression in NK cells through an NKG2D dependent pathway, implicating its potential to increase Perforin expression by NKG2D expressing GD T cells as well (82). Given the deleterious effects of Perforin expressing cells in the context of autoimmune and inflammatory disorders, blocking therapeutics targeting Perforin may benefit patients suffering from these illnesses. Compounds identified as Perforin inhibitors in NK and AB T cells include diarylthiophenes and benzenesulfonamide based therapeutics (83–85). In short, cutaneous GD T cells express Perforin upon activation and this activation can be achieved through a variety of mechanisms including stimulation of the TCR, ligation of costimulatory molecules, and cytokine stimulation (Table 1). The critical role of Perforin in executing GD T cell mediated cytotoxicity in the skin warrants further studies on the mechanisms that induce Perforin expression by these cells.

## Perforin-2 Expression by GD T Cells

Perforin-2 is a recently identified member of the MACPF domain containing pore forming family of innate immune proteins that plays a critical role in clearance of intracellular bacterial infections. However, the mechanisms behind Perforin-2 activation and the extent of its contributions to host immunity have not been fully characterized. Our group was the first to report Perforin-2 expression in a variety of cell types in human skin including keratinocytes, fibroblasts and GD T cells (68). Although it is known that both murine and human GD T cells express Perforin-2, the role that it plays in GD T cell mediated cytotoxicity toward bacterial pathogens is still unknown. We demonstrated that Perforin-2 expression is upregulated in CD45+ cells upon wounding in an *ex vivo* human skin model. However, infection of the wounds with *Staphylococcus aureus* inhibited Perforin-2 expression in these cells (68). This indicates a potential role for Perforin-2 in promoting skin homeostasis and barrier integrity by aiding in the clearance of bacteria from the wound site (Figure 1C). This was confirmed by our finding that human keratinocyte cells constitutively expressing a Perforin-2-GFP fusion protein demonstrate improved clearance of intracellular *S. aureus* infection in comparison to control cells (68). Our group has also demonstrated that Perforin-2 deficient mice infected epicutaneously are unable to clear *S. aureus* and eventually succumb to the infection (48). Interestingly, in this issue we provide evidence that *S. epidermidis*, a skin commensal microorganism, induces Perforin-2 in GD T cells in human skin *ex vivo* (66). Given the established role of GD T cells in the antimicrobial response and in keratinocyte proliferation and migration upon wounding, it is likely that disruption of the epidermal barrier and signals from microorganisms can result in Perforin-2 induction. This warrants further studies on Perforin-2 regulation and its function as an effector protein in GD T cell mediated skin immune responses.

## Conclusions

Maintaining skin homeostasis and barrier function is essential for protection against physical and chemical stress, infections, and malignancies. Recent research has highlighted the significant contributions of GD T cells to skin health through their role in bacterial clearance, wound healing, and tumor killing. Expression of both Perforin and Perforin-2 by GD T cells has been implicated in the activation of these effector functions (Figure 1, Table 1). GD T cells lyse a variety of tumor cell lines and exhibit many characteristics of conventional cytotoxic T cells. Although GD T cells have been shown to produce Perforin, little is known about the signals that stimulate its expression. Additionally, Perforin-2 was recently identified as another member of the MACPF domain containing pore forming protein family. It is critical for clearance of intracellular bacterial infections, and it is expressed constitutively by GD T cells. Given the established role of GD T cells as first responders to bacterial infections in the skin, it is important to further investigate the regulation of Perforin-2 in this GD T cell function. Understanding the signals that activate Perforin expression by GD T cells can help to elucidate the mechanisms governing their diverse roles in skin immunity.

## Author Contributions

KO'N wrote the manuscript and prepared the figures. IP, MT-C, and NS revised the manuscript and provided critical comments. All authors contributed to the article and approved the submitted version.

## Conflict of Interest

The authors declare that the research was conducted in the absence of any commercial or financial relationships that could be construed as a potential conflict of interest.

## References

[1] Chien Y Meyer C Bonneville M. γδ T cells: first line of defense and beyond. Annu Rev Immunol. (2014) 32:121–55. 10.1146/annurev-immunol-032713-120216 24387714

[2] Havran WL Jameson JM Witherden DA. Epithelial cells and their neighbors. III. Interactions between intraepithelial lymphocytes and neighboring epithelial cells. Am J Physiol Liver Physiol. (2005) 289:G627–30. 10.1152/ajpgi.00224.2005 16160077

[3] Groh V Porcelli S Fabbi M Lanier LL Picker LJ Anderson T. Human lymphocytes bearing T cell receptor gamma/delta are phenotypically diverse and evenly distributed throughout the lymphoid system. J Exp Med. (1989) 169:1277–94. 10.1084/jem.169.4.1277 2564416 PMC2189233

[4] Parker CM Groh V Band H Porcelli SA Morita C Fabbi M. Evidence for extrathymic changes in the T cell receptor gamma/delta repertoire. J Exp Med. (1990) 171:1597–612. 10.1084/jem.171.5.1597 2185330 PMC2187908

[5] Cai Y Shen X Ding C Qi C Li K Li X. Pivotal role of dermal IL-17-producing γδ T cells in skin inflammation. Immunity. (2011) 35:596–610. 10.1016/j.immuni.2011.08.001 21982596 PMC3205267

[6] Gray EE Suzuki K Cyster JG. Cutting edge: identification of a motile IL-17-producing gammadelta T cell population in the dermis. J Immunol. (2011) 186:6091–5. 10.4049/jimmunol.1100427 21536803 PMC3098921

[7] Itohara S Farr AG Lafaille JJ Bonneville M Takagaki Y Haas W. Homing of a γδ thymocyte subset with homogeneous T-cell receptors to mucosal epithelia. Nature. (1990) 343:754–7. 10.1038/343754a0 2154700

[8] Goodman T Lefrancois L. Intraepithelial lymphocytes. Anatomical site, not T cell receptor form, dictates phenotype and function. J Exp Med. (1989) 170:1569–81. 10.1084/jem.170.5.1569 2572671 PMC2189511

[9] Laggner U Di Meglio P Perera GK Hundhausen C Lacy KE Ali N. Identification of a novel proinflammatory human skin-homing Vγ9Vδ2 T cell subset with a potential role in psoriasis. J Immunol. (2011) 187:2783–93. 10.4049/jimmunol.1100804 21813772 PMC3187621

[10] Witherden DA Havran WL. Cross-talk between intraepithelial γδ T cells and epithelial cells. J Leukoc Biol. (2013) 94:69–76. 10.1189/jlb.0213101 23620015 PMC3685022

[11] Whang MI Guerra N Raulet DH. Costimulation of dendritic epidermal γδ T cells by a new NKG2D ligand expressed specifically in the skin. J Immunol. (2009) 182:4557–64. 10.4049/jimmunol.0802439 19342629 PMC3001286

[12] Bonneville M O'Brien RL Born WK. γδ T cell effector functions: a blend of innate programming and acquired plasticity. Nat Rev Immunol. (2010) 10:467–78. 10.1038/nri2781 20539306

[13] Strid J Sobolev O Zafirova B Polic B Hayday A. The intraepithelial T cell response to NKG2D-ligands links lymphoid stress surveillance to atopy. Science. (2011) 334:1293–7. 10.1126/science.1211250 22144628 PMC3842529

[14] Havran WL Allison JP. Origin of Thy-1+ dendritic epidermal cells of adult mice from fetal thymic precursors. Nature. (1990) 344:68–70. 10.1038/344068a0 1968230

[15] Bos JD Teunissen MBM Cairo I Krieg SR Kapsenberg ML Das PK. T-Cell receptor γδ bearing cells in normal human skin. J Invest Dermatol. (1990) 94:37–42. 10.1111/1523-1747.ep12873333 1688597

[16] Clark RA Chong B Mirchandani N Brinster NK Yamanaka K-I Dowgiert RK. The vast majority of CLA+ T cells are resident in normal skin. J Immunol. (2006) 176:4431–9. 10.4049/jimmunol.176.7.4431 16547281

[17] Alaibac M Morris J Yu R Chu AC. T lymphocytes bearing the γδ T-cell receptor: a study in normal human skin and pathological skin conditions. Br J Dermatol. (1992) 127:458–62. 10.1111/j.1365-2133.1992.tb14840.x 1467283

[18] Ebert LM Meuter S Moser B. Homing and function of human skin gammadelta T cells and NK cells: relevance for tumor surveillance. J Immunol. (2006) 176:4331–6. 10.4049/jimmunol.176.7.4331 16547270

[19] Cruz MS Diamond A Russell A Jameson JM. Human αβ and γδ T cells in skin immunity and disease. Front Immunol. (2018) 9:1304. 10.3389/fimmu.2018.01304 29928283 PMC5997830

[20] Vantourout P Hayday A. Six-of-the-best: unique contributions of γδ T cells to immunology. Nat Rev Immunol. (2013) 13:88–100. 10.1038/nri3384 23348415 PMC3951794

[21] Morita CT Lee HK Leslie DS Tanaka Y Bukowski JF Märker-Hermann E Recognition of nonpeptide prenyl pyrophosphate antigens by human γδ T cells. Microbes Infect. (1999) 1:175–86. 10.1016/S1286-4579(99)80032-X 10594981

[22] Sumaria N Roediger B Ng LG Qin J Pinto R Cavanagh LL. Groth B, Triccas JA, Weninger W. Cutaneous immunosurveillance by self-renewing dermal γδ T cells. J Exp Med. (2011) 208:505–18. 10.1084/jem.20101824 21339323 PMC3058585

[23] Pantelyushin S Haak S Ingold B Kulig P Heppner FL Navarini AA. Rorγt+ innate lymphocytes and γδ T cells initiate psoriasiform plaque formation in mice. J Clin Invest. (2012) 122:2252–6. 10.1172/JCI61862 22546855 PMC3366412

[24] Toulon A Breton L Taylor KR Tenenhaus M Bhavsar D Lanigan C. A role for human skin–resident T cells in wound healing. J Exp Med. (2009) 206:743–50. 10.1084/jem.20081787 19307328 PMC2715110

[25] Jameson J Ugarte K Chen N Yachi P Fuchs E Boismenu R A role for skin gamma delta T cells in wound repair. Science. (2002) 296:747–9. 10.1126/science.1069639 11976459

[26] MacLeod AS Hemmers S Garijo O Chabod M Mowen K Witherden DA. Dendritic epidermal T cells regulate skin antimicrobial barrier function. J Clin Invest. (2013) 123:4364–74. 10.1172/JCI70064 24051381 PMC3784546

[27] Gay D Kwon O Zhang Z Spata M Plikus M V Holler PD. Fgf9 from dermal γδ T cells induces hair follicle neogenesis after wounding. Nat Med. (2013) 19:916–23. 10.1038/nm.3181 23727932 PMC4054871

[28] Kaminski MJ Cruz PD Bergstresser PR Takashima A. Killing of skin-derived tumor cells by mouse dendritic epidermal T-cells. Cancer Res. (1993) 53:4014–9. 8358730

[29] Girardi M Oppenheim DE Steele CR Lewis JM Glusac E Filler R Regulation of cutaneous malignancy by gamma delta T Cells. Science. (2001) 294:605–9. 10.1126/science.1063916 11567106

[30] Rosado CJ Kondos S Bull TE Kuiper MJ Law RHP Buckle AM. The MACPF/CDC family of pore-forming toxins. Cell Microbiol. (2008) 10:1765–74. 10.1111/j.1462-5822.2008.01191.x 18564372 PMC2654483

[31] Gilbert RJC. Cholesterol-dependent cytolysins. Adv Exp Med Biol. (2010) 677:56–66. 10.1007/978-1-4419-6327-7_5 20687480

[32] Tweten RK. Cholesterol-dependent cytolysins, a family of versatile pore-forming toxins. Infect Immun. (2005) 73:6199–209. 10.1128/IAI.73.10.6199-6209.2005 16177291 PMC1230961

[33] Hadders MA Beringer DX Gros P. Structure of C8α-MACPF reveals mechanism of membrane attack in complement immune defense. Science. (2007) 317:1552–4. 10.2210/pdb2qqh/pdb 17872444

[34] Rosado CJ Buckle AM Law RHP Butcher RE Kan WT Bird CH. A common fold mediates vertebrate defense and bacterial attack. Science. (2007) 317:1548–51. 10.1126/science.1144706 17717151

[35] Slade DJ Lovelace LL Chruszcz M Minor W Lebioda L Sodetz JM Crystal structure of the MACPF domain of human complement protein C8α in complex with the C8γ subunit. J Mol Biol. (2008) 379:331–42. 10.1016/j.jmb.2008.03.061 18440555 PMC2443722

[36] Tschopp J Masson D Stanley KK. Structural/functional similarity between proteins involved in complement- and cytotoxic T-lymphocyte-mediated cytolysis. Nature. (1986) 322:831–4. 10.1038/322831a0 2427956

[37] Lovelace LL Cooper CL Sodetz JM Lebioda L. Structure of human C8 protein provides mechanistic insight into membrane pore formation by complement. J Biol Chem. (2011) 286:17585–92. 10.1074/jbc.M111.219766 21454577 PMC3093833

[38] Kondos SC Hatfaludi T Voskoboinik I Trapani JA Law RHP Whisstock JC. The structure and function of mammalian membrane-attack complex/perforin-like proteins. Tissue Antigens. (2010) 76:341–51. 10.1111/j.1399-0039.2010.01566.x 20860583

[39] Podack ER Young JDE Cohn ZA. Isolation and biochemical and functional characterization of perforin 1 from cytolytic T-cell granules. Proc Natl Acad Sci USA. (1985) 82:8629–33. 10.1073/pnas.82.24.8629 2417226 PMC390971

[40] Lopez JA Susanto O Jenkins MR Lukoyanova N Sutton VR Law RHP. Perforin forms transient pores on the target cell plasma membrane to facilitate rapid access of granzymes during killer cell attack. Blood. (2013) 121:2659–68. 10.1182/blood-2012-07-446146 23377437

[41] Leung C Hodel AW Brennan AJ Lukoyanova N Tran S House CM. Real-time visualization of perforin nanopore assembly. Nat Nanotechnol. (2017) 12:467–73. 10.1038/nnano.2016.303 28166206

[42] Benard EL Racz PI Rougeot J Nezhinsky AE Verbeek FJ Spaink HP. Macrophage-expressed perforins mpeg1 and mpeg1.2 have an anti-bacterial function in zebrafish. J Innate Immun. (2015) 7:136–52. 10.1159/000366103 25247677 PMC6738794

[43] Wiens M Korzhev M Krasko A Thakur NL Perović-Ottstadt S Breter HJ. Innate immune defense of the sponge *suberites domuncula* against bacteria involves a myD88-dependent signaling pathway. J Biol Chem. (2005) 280:27949–59. 10.1074/jbc.M504049200 15923643

[44] McCormack R Podack ER. Perforin-2/Mpeg1 and other pore-forming proteins throughout evolution. J Leukoc Biol. (2015) 98:761–8. 10.1189/jlb.4MR1114-523RR 26307549 PMC4600061

[45] D'Angelo ME Dunstone MA Whisstock JC Trapani JA Bird PI. Perforin evolved from a gene duplication of MPEG1, followed by a complex pattern of gene gain and loss within luteleostomi. BMC Evol Biol. (2012) 12:59. 10.1186/1471-2148-12-59 22551122 PMC3477005

[46] Ni T Jiao F Yu X Aden S Ginger L Williams SI. Structure and mechanism of bactericidal mammalian perforin-2, an ancient agent of innate immunity. Sci Adv. (2020) 6:eaax8286. 10.1126/sciadv.aax8286 32064340 PMC6989145

[47] Pang SS Bayly-Jones C Radjainia M Spicer BA Law RHP Hodel AW. The cryo-EM structure of the acid activatable pore-forming immune effector macrophage-expressed gene 1. Nat Commun. (2019) 10:4288. 10.1038/s41467-019-12279-2 31537793 PMC6753088

[48] McCormack RM de Armas LR Shiratsuchi M Fiorentino DG Olsson ML Lichtenheld MG. Perforin-2 is essential for intracellular defense of parenchymal cells and phagocytes against pathogenic bacteria. Elife. (2015) 4:e508. 10.7554/eLife.06508 26402460 PMC4626811

[49] McCormack R de Armas LR Shiratsuchi M Ramos JE Podack ER. Inhibition of intracellular bacterial replication in fibroblasts is dependent on the perforin-like protein (perforin-2) encoded by macrophage-expressed gene 1. J Innate Immun. (2013) 5:185–94. 10.1159/000345249 23257510 PMC3732477

[50] Smyth MJ Ortaldo JR Shinkaij YI Yagita H Nakata M Okumura K. Interleukin 2 induction of pore-forming protein gene expression in human peripheral blood CD8+ T cells. J Exp Med. (1990) 171:1269–81. 10.1084/jem.171.4.1269 1691263 PMC2187847

[51] Krähenbühl O Gattesco S Tschopp J. Murine Thy-1+ dendritic epidermal t cell lines express granule-associated perforin and a family of granzyme molecules. Immunobiology. (1992) 184:392–401. 10.1016/S0171-2985(11)80596-6 1350566

[52] Kägi D Ledermann B Bürki K Zinkernagel RM Hengartner H. Molecular mechanisms of lymphocyte-mediated cytotoxicity and their role in immunological protection and pathogenesis *in vivo*. Annu Rev Immunol. (1996) 14:207–32. 10.1146/annurev.immunol.14.1.207 8717513

[53] Nitahara A Shimura H Ito A Tomiyama K Ito M Kawai K. NKG2D ligation without T cell receptor engagement triggers both cytotoxicity and cytokine production in dendritic epidermal T Cells. J Invest Dermatol. (2006) 126:1052–8. 10.1038/sj.jid.5700112 16484989

[54] Campillo JA Martínez-Escribano JA Minguela A López-Álvarez R Marín LA García-Alonso AM. Increased number of cytotoxic CD3+CD28- γδ T cells in peripheral blood of patients with cutaneous malignant melanoma. Dermatology. (2007) 214:283–8. 10.1159/000100878 17460398

[55] Wakita D Sumida K Iwakura Y Nishikawa H Ohkuri T Chamoto K. Tumor-infiltrating IL-17-producing γδ T cells support the progression of tumor by promoting angiogenesis. Eur J Immunol. (2010) 40:1927–37. 10.1002/eji.200940157 20397212

[56] Toro JR Beaty M Sorbara L Turner ML White J Kingma DW. γδ T-cell lymphoma of the skin. Arch Dermatol. (2000) 136:1024–32. 10.1001/archderm.136.8.1024 10926739

[57] Rodríguez-Pinilla SM Ortiz-Romero PL Monsalvez V Tomás IE Almagro M Sevilla A. TCR-γ expression in primary cutaneous T-cell lymphomas. Am J Surg Pathol. (2013) 37:375–84. 10.1097/PAS.0b013e318275d1a2 23348211

[58] Salhany KE Macon WR Choi JK Elenitsas R Lessin SR Felgar RE. Subcutaneous panniculitis-like T-cell lymphoma. Am J Surg Pathol. (1998) 22:881–93. 10.1097/00000478-199807000-00010 9669350

[59] Boulland ML Wechsler J Bagot M Pulford K Kanavaros P Gaulard P. Primary CD30-positive cutaneous T-cell lymphomas and lymphomatoid papulosis frequently express cytotoxic proteins. Histopathology. (2000) 36:136–44. 10.1046/j.1365-2559.2000.00799.x 10672058

[60] Ibusuki A Kawai K Yoshida S Uchida Y Nitahara-Takeuchi A Kuroki K. NKG2D triggers cytotoxicity in murine epidermal γδ T cells via PI3K-dependent, Syk/ZAP70-independent signaling pathway. J Invest Dermatol. (2014) 134:396–404. 10.1038/jid.2013.353 23962808

[61] Schuhmachers G Ariizumi K Mathew PA Bennett M Kumar V Takashima A. 2B4, a new member of the immunoglobulin gene superfamily, is expressed on murine dendritic epidermal T cells and plays a functional role in their killing of skin tumors. J Invest Dermetol. (1995). 105:592–6. 10.1111/1523-1747.ep12323533 7561164

[62] Yeh K-Y Zhong C Nasir A Ohsuga Y Takashima A Lord EM. Expression of B7–1 by pam 212 squamous cell carcinoma enhances tumor cell interactions with dendritic epidermal t cells but does not affect *in vivo* tumor growth. J Invest Dermatol. (1997). 109:728–33. 10.1111/1523-1747.ep12340723 9406812

[63] De Creus A Van Beneden K Stevenaert F Debacker V Plum J Leclercq G. Developmental and functional defects of thymic and epidermal V gamma 3 cells in IL-15-deficient and IFN regulatory factor-1-deficient mice. J Immunol. (2002) 168:6486–93. 10.4049/jimmunol.168.12.6486 12055269

[64] Hirsh MI Junger WG. Roles of heat shock proteins and gamma delta T cells in inflammation. Am J Respir Cell Mol Biol. (2008) 39:509–13. 10.1165/rcmb.2008-0090TR 18566334 PMC2574523

[65] Dao V Liu Y Pandeswara S Svatek RS Gelfond JA Liu A. Immune-stimulatory effects of rapamycin are mediated by stimulation of antitumor γδ T cells. Cancer Res. (2016) 76:5970–82. 10.1158/0008-5472.CAN-16-0091 27569211 PMC5065775

[66] Strbo N O'Neill KE Head CR Padula L Stojadinovic O Pastar I *Staphylococcus epidermidis* facilitates intracellular pathogen clearance through upregulation of antimicrobial protein perforin-2 (P-2) in the human skin gamma delta T cells. J Immunol. (2020) 204(1 Supplement):157.10.

[67] Van Beneden K De Creus A Stevenaert F Debacker V Plum J Leclercq G. Expression of inhibitory receptors ly49e and cd94/nkg2 on fetal thymic and adult epidermal tcr vγ3 lymphocytes. J Immunol. (2002) 168:3295–302. 10.4049/jimmunol.168.7.3295 11907085

[68] Strbo N Pastar I Romero L Chen V Vujanac M Sawaya AP. Single cell analyses reveal specific distribution of anti-bacterial molecule perforin-2 in human skin and its modulation by wounding and *Staphylococcus aureus* infection. Exp Dermatol. (2019) 28:225–32. 10.1111/exd.13870 30609079 PMC7461719

[69] Giacomelli R Cipriani P Fulminis A Barattelli G Matucci-Cerinic M D'Alo S. Circulating gamma/delta T lymphocytes from systemic sclerosis (SSc) patients display a T helper (Th) 1 polarization. Clin Exp Immunol. (2001) 125:310–5. 10.1046/j.1365-2249.2001.01603.x 11529924 PMC1906121

[70] Giacomelli R Matucci-Cerinic M Cipriani P Ghersetich I Lattanzio R Pavan A. Circulating Vδ1 + T cells are activated and accumulate in the skin of systemic sclerosis patients. Arthritis Rheum. (1998) 41:327–34. 10.1002/1529-013141:2<327::AID-ART17>3.0.CO;2-S 9485091

[71] Henriques A Silva C Santiago M Henriques MJ Martinho A Trindade H. Subset-specific alterations in frequencies and functional signatures of γδ T cells in systemic sclerosis patients. Inflamm Res. (2016) 65:985–94. 10.1007/s00011-016-0982-6 27576328

[72] Das D Anand V Khandpur S Sharma VK Sharma A. T helper type 1 polarizing γδ T cells and scavenger receptors contribute to the pathogenesis of pemphigus vulgaris. Immunology. (2018) 153:97–104. 10.1111/imm.12814 28815581 PMC5721249

[73] Accardo-Palumbo A Giardina AR Ciccia F Ferrante A Principato A Impastato R. Phenotype and functional changes of Vγ9/Vδ2 T lymphocytes in behçet's disease and the effect of infliximab on Vγ9/Vδ2 T cell expansion, activation and cytotoxicity. Arthritis Res Ther. (2010) 12:R109. 10.1186/ar3043 20525258 PMC2911900

[74] Cañete JD Celis R Noordenbos T Moll C Gómez-Puerta JA Pizcueta P. Distinct synovial immunopathology in Behçet disease and psoriatic arthritis. Arthritis Res Ther. (2009) 11:R17. 10.1186/ar2608 19196489 PMC2688249

[75] Kastelan M Prpic Massari L Gruber F Zamolo G Zauhar G Coklo M. Perforin expression is upregulated in the epidermis of psoriatic lesions. Br J Dermatol. (2004) 151:831–6. 10.1111/j.1365-2133.2004.06168.x 15491424

[76] Prpić Massari L Kaštelan M Laškarin G Zamolo G Massari D Rukavina D. Analysis of perforin expression in peripheral blood and lesions in severe and mild psoriasis. J Dermatol Sci. (2007) 47:29–36. 10.1016/j.jdermsci.2007.02.007 17412565

[77] Yawalkar N Schmid S Braathen LR Pichler WJ. Perforin and granzyme B may contribute to skin inflammation in atopic dermatitis and psoriasis. Br J Dermatol. (2001) 144:1133–9. 10.1046/j.1365-2133.2001.04222.x 11422032

[78] Polese B Zhang H Thurairajah B King IL. Innate lymphocytes in psoriasis. Front Immunol. (2020) 11:242. 10.3389/fimmu.2020.00242 32153574 PMC7047158

[79] Bousso P Robey E. Dynamics of CD8+ T cell priming by dendritic cells in intact lymph nodes. Nat Immunol. (2003) 4:579–85. 10.1038/ni928 12730692

[80] Maasho K Opoku-Anane J Marusina AI Coligan JE Borrego F. Cutting edge: NKG2D Is a costimulatory receptor for human naive CD8 + T Cells. J Immunol. (2005) 174:4480–4. 10.4049/jimmunol.174.8.4480 15814668

[81] Bauer S Groh V Wu J Steinle A Phillips JH Lanier LL Activation of NK cells and T cells by NKG2D, a receptor for stress- inducible MICA. Science. (1999) 174:4480–4. 10.1126/science.285.5428.727 10426993

[82] Lu C-C Chen J-K. Resveratrol enhances perforin expression and NK cell cytotoxicity through NKG2D-dependent pathways. J Cell Physiol. (2010) 223:343–51. 10.1002/jcp.22043 20082299

[83] Miller CK Huttunen KM Denny WA Jaiswal JK Ciccone A Browne KA. Diarylthiophenes as inhibitors of the pore-forming protein perforin. Bioorg Med Chem Lett. (2016) 26:355. 10.1016/j.bmcl.2015.12.003 26711151 PMC4706532

[84] Spicer JA Miller CK O'Connor PD Jose J Huttunen KM Jaiswal JK. Benzenesulphonamide inhibitors of the cytolytic protein perforin. Bioorg Med Chem Lett. (2017) 27:1050–4. 10.1016/j.bmcl.2016.12.057 28110869 PMC5303009

[85] Welz M Eickhoff S Abdullah Z Trebicka J Gartlan KH Spicer JA. Perforin inhibition protects from lethal endothelial damage during fulminant viral hepatitis. Nat Commun. (2018) 9:4805. 10.1038/s41467-018-07213-x 30442932 PMC6237769

